# Supplementary material for: Super-Sensitive LC-MS Analyses of Exposure Biomarkers for Multiple Mycotoxins in a Rural Pakistan Population
Source: Toxins (Basel). 2022 Mar 4;14(3):193. doi: 10.3390/toxins14030193 (PMC8950642; doi:10.3390/toxins14030193)
Supplement: Supplementary file 1 [file toxins-14-00193-s001.zip › toxins-1590102-supplementary.pdf]

Supplementary Materials: Super-Sensitive LC-MS Analyses of Exposure Biomarkers for Multiple Mycotoxins in a Rural Pakistan Population

Lei Xia [1], Hifza Rasheed [2], Michael N. Routledge [3,4], Hang Wu [3] and Yun Yun Gong [1,*]

Matrix effect evaluation:

The matrix effect for the rice and wheat samples were calculated by the equation below:

$$Matrix\ effect\ (\%) = \frac{Average\ area\ of\ post\ spiked\ samples}{Average\ area\ of\ solvent\ standards} \times 100\%$$

The post spiked samples were prepared by spiking mycotoxin standards into the extractants from the blank rice/wheat/urine samples. For rice and wheat samples, three different concentrations (low, medium and high) were used to evaluate the matrix effect, whereas for urine samples, concentrations matched with the standard curve were used. The results are summarized in Table S1, the results shown are from three repeats.

Table S1. Matrix effect of rice, wheat and urine on screened analytes.

Analyte	Matrix effect (*n* = 3)	Analyte	Matrix effect (*n* = 3)	Analyte	Matrix effect (*n* = 3)
Rice_AFB_1	87	Wheat_AFB_1	87	Urine_AFM_1	79
Rice_DON	81	Wheat_DON	84	Urine_DON	77
Rice_FB_1	91	Wheat_FB_1	85	Urine_DOM-1	98
Rice_OTA	92	Wheat_OTA	93	Urine_FB_1	96
Rice_ZEN	101	Wheat_ZEN	103	Urine_OTA	99
				Urine_ZEN	87
				Urine_α-ZEL	83
				Urine_β-ZEL	82

Food UPLC-MS/MS analysis parameters

The sample (10 μL) was injected on a Waters Acquity UPLC BEH C18 column (2.1 × 50 mm, 1.7 μm particle size) operated at 40 °C. The solvent gradient began with 90% mobile phase (A) 0.1% formic acid in water and 10% mobile phase (B) methanol; reached 100% B over 15 min; the system was retained at 100% B for 3 min, followed by 10% B to equilibrate the system for 2 minutes. The flow rate was 0.35 mL/min. The MS was operating in positive mode with 3.35 kV and negative mode with -2.5 kV spray voltage, 50 Arb sheath gas, 15 Arb auxiliary gas, 2 Arb sweep gas, the ion transfer tube was set at a temperature of 350 °C and the vaporization temperature was 400 °C.

Table S2. Optimised MS and MS/MS parameters for mycotoxin in rice/wheat samples.

Analyte	Retent ion time (min)	Parent ion (m z^{-1})	Ion species	Product ion (m z^{-1})	CE (V)	RF lens (V)
**DON**	2.28	297	$[M+H]^+$	249 (Qf)<br>231 (Ql)	10<br>11	37
**AFB_1**	6.93	313	$[M+H]^+$	241 (Qf)<br>285 (Ql)	38<br>30	125
**FB_1**	9.20	722	$[M+H]^+$	334 (Qf)<br>352 (Ql)	42<br>32	127
**OTA**	10.12	404	$[M+H]^+$	239 (Qf)<br>221 (Ql)	23<br>33	61
**ZEN**	9.84	317	$[M-H]^-$	175 (Qf)<br>131 (Ql)	−24<br>−27	83

Qf: quantification ion; Ql: qualification ion; CE: collision energy .

Urine UPLC-MS/MS analysis parameters

The sample (8 μL) was injected on a Waters Acquity UPLC CORTECS C18 column (2.1 × 100 mm, 2.1 μm particle size) operated at 40 °C. The solvent gradient began with 90% mobile phase (A) 0.1% formic acid in water and 10% mobile phase (B) ACN:methanol (3:7, v/v); reached 50% B over 4 min and then reached 100% B over 10 mins; the system was retained at 100% B for 5 min, followed by 10% B to equilibrate the system for 5 minutes. The flow rate was 0.35 mL/min. The MS was operating in positive mode with 3kV and negative mode with −0.5 kV spray voltage, with source temperature of 150 °C, desolvation temperature 500 °C, cone gas flow 150 L/hr, desolvation gas flow 1000 L/hr, collision gas flow 0.15 mL/min and under 7 bar of nebulizer gas flow.

Table S3. Optimised MS and MS/MS parameters for mycotoxin urinary biomarkers.

Analyte	Retention time (min)	Cone voltage (V)	Parent ion (m z^{-1})	Ion species	Product ion (m z^{-1})	CE (eV)
DON	2.54	10	297	$[M+H]^+$	249 (Qf)<br>231 (Ql)	12<br>12
$^{13}C_{15}$-DON	2.54	10	312	$[M+H]^+$	263 (Qf)<br>216 (Ql)	10<br>14
DOM-1	3.39	22	281	$[M+H]^+$	233 (Qf)<br>109 (Ql)	10<br>14
AFM_1	4.91	8	329	$[M+H]^+$	273 (Qf)<br>259 (Ql)	22<br>24
$^{13}C_{17}$-AFM_1	4.91	8	346	$[M+H]^+$	288 (Qf)<br>242 (Ql)	28<br>38
FB_1	6.48	32	722	$[M+H]^+$	334 (Qf)<br>352 (Ql)	34<br>34
$^{13}C_{34}$-FB_1	6.48	32	756	$[M+H]^+$	356 (Qf)<br>374 (Ql)	42<br>36
OTA	9.58	20	404	$[M+H]^+$	239 (Qf)<br>221 (Ql)	14<br>24
$^{13}C_{20}$-OTA	9.58	20	424	$[M+H]^+$	377 (Qf)<br>250 (Ql)	12<br>24
ZEN	9.01	42	317	$[M-H]^-$	175 (Qf)<br>131 (Ql)	−22<br>−30
α-ZEL	8.12	48	319	$[M-H]^-$	160 (Qf)<br>174 (Ql)	−32<br>−26
β-ZEL	7.05	44	319	$[M-H]^-$	160 (Qf)<br>174 (Ql)	−32<br>−26

Qf: quantification ion; Ql: qualification ion; CE: collision energy.
